# Fusion of Computational Intelligence Techniques and Their Practical Applications

**DOI:** 10.1155/2015/463147

**Published:** 2015-08-30

**Authors:** Rahib H. Abiyev, Rafik Aliev, Okyay Kaynak, I. Burhan Turksen, Karl Walter Bonfig

**Affiliations:** ^1^Faculty of Engineering, Applied Artificial Intelligence Research Centre Near East University, Lefkoşa, North Cyprus, Mersın 10, Turkey; ^2^Department of Computer-Aided Control System, Azerbaijan State Oil Academy, Azadlik Avenue 20, Baku, Azerbaijan; ^3^Department of Electrical and Electronic Engineering, Bogazici University, Bebek, 80815 Istanbul, Turkey; ^4^University of Toronto, 170 College Street (Rear), Haultain Building, Room 305A, Toronto, ON, Canada M5S 3G8; ^5^Department of Electrical Engineering and Information, University of Siegen, 57068 Siegen, Germany

Computational intelligence techniques inspired by evolution, by nature, and by the brain are playing an important role in the solution of complex real-world problems. Fusion of computational intelligence techniques integrates neural networks, fuzzy systems, and evolutionary computing into a system design that enables handling of complexity and managing of uncertainty and imprecision. Each respective technique enhances the capability of the other and the fusion of these paradigms in system design offsets the demerits of one paradigm by the merits of another. Recently, computational intelligence techniques have been widely applied to a wide variety of complex problems, including engineering, science, and business. However, due to complexity and uncertainty in these problems, it becomes difficult to find out the optimal solution of the problems. Hereby it is necessary to consider the latest trends and developments in the field of fusion of computational intelligence techniques and to develop efficient computational models for solving practical problems. Fusion of computational intelligence techniques covers the spectrum of applications, comprehensively demonstrating the advantages of fusion techniques in industrial applications that deal with various kinds of inaccuracies and uncertainties.

The aim of this special issue was the presentation of research articles as well as review articles incorporating the contributions in theories, the structures, algorithms, and advances in the design of system based on fusion of neural networks, fuzzy systems, and evolutionary algorithms and their practical applications.

The following is a brief summary for each of the accepted articles.

The paper “A Method for Estimating View Transformations from Image Correspondences Based on the Harmony Search Algorithm” by E. Cuevas and M. Díaz presents a new improved algorithm that combines the random sampling consensus (RANSAC) method and the Harmony Search (HS) algorithm for estimation of the model parameters from a data set. The proposed algorithm adopts a different sampling strategy than RANSAC to generate putative solutions and can substantially reduce the number of learning iterations. At each iteration, new candidate solutions are generated iteratively by taking into consideration the quality of models produced by previous candidate solutions. The rules for the generation of candidate solutions are motivated by the improvisation process that occurs when a musician searches for a better state of harmony. The method is used for the estimation of homographies, considering synthetic and real images, and it is also employed for position estimation in a humanoid robot.

The paper “Application of *Z*-Number Based Modeling in Psychological Research” by R. Aliev and K. Memmedova uses *Z*-number based fuzzy approach for modelling the effect of Pilates exercises on motivation, attention, anxiety, and educational achievement of students. The grade point average of the students was used as the measure of educational achievement. The inference techniques for approximate reasoning based on *Z*-interpolation method suggested by Zadeh are used in the decision making process. The basic steps of *Z*-number based modelling with numerical solutions are presented.

The paper “Fusing Swarm Intelligence and Self-Assembly for Optimizing Echo State Networks” by C. E. Martin and J. A. Reggia considers the optimizing of a neural network's weights and topology using integration of self-assembly (SA) and particle swarm optimization (PSO). The authors developed a model that integrates network self-assembly and particle swarm optimization for the purpose of growing neural networks with weights and topologies that are optimized for specified computational tasks. The presented model is used for optimizing echo state network weights and topologies on a number of challenging benchmark problems from the domains of time-series forecasting and control.

The paper “Cuckoo Search Algorithm Based on Repeat-Cycle Asymptotic Self-Learning and Self-Evolving Disturbance for Function Optimization” by J. Wang et al. developed a new improved cuckoo search algorithm based on the repeat-cycle asymptotic self-learning and self-evolving disturbance (RC-SSCS). A disturbance operation is added to the algorithm in order to make a more careful search near the bird's nests location. The repeat-cycle asymptotic mode is to narrow the disturbance scope based on the last disturbance results and then go on the next disturbance. The proposed algorithm improves convergence velocity and optimization accuracy of the cuckoo search (CS) algorithm for solving the function optimization problems. This improved algorithm overcomes the CS algorithm's defects which result from its high degree of random and strong leap and also makes full use of the information near the bird's nest location that had been found. The comparative results with the benchmarking functions show that the improved cuckoo search algorithm has better convergence velocity and optimization accuracy.

The paper “Symmetry Based Automatic Evolution of Clusters: A New Approach to Data Clustering” by S. Vijendra and S. Laxman presents a multiobjective genetic clustering approach, in which data points are assigned to clusters based on new line symmetry distance. The proposed multiobjective line symmetry based genetic clustering (MOLGC) algorithm evolves near-optimal clustering solutions using multiple clustering criteria, without a priori knowledge of the actual number of clusters. The multiple randomized dimensional trees based nearest neighbour search is used to reduce the complexity of finding the closest symmetric points. Experimental results show that proposed algorithm can obtain optimal clustering solutions in terms of different cluster quality measures.

The paper “Optimization of High-Dimensional Functions through Hypercube Evaluation” by R. H. Abiyev and M. Tunay proposes a novel evolutionary learning algorithm based on evaluation and optimization of a hypercube for solving global numerical optimization problems. The algorithm is inspired from the behaviour of doves discovering new areas for food in natural life. The HO algorithm comprises the initialization and evaluation process, displacement-shrink process, and searching space process. The initialization and evaluation process initializes initial solution and evaluates the solutions in given hypercube. The displacement-shrink process determines displacement and evaluates objective functions using new points; the search area process determines next hypercube using certain rules and evaluates the new solutions. The HO algorithm is tested on a set of specific benchmarking functions and has shown better performance for global optimization of both low- and high-dimensional problems with large numbers of local optimal.

The paper “A Simple Fitness Function for Minimum Attribute Reduction” by Y. Su et al. considers the problem of finding the minimal subset of the condition attribute set such that minimal set has the same classification quality as the condition attribute set. For this purpose, the design of fitness function that satisfies the equivalence between the optimal solution and the minimal attribute reduction is considered. The optimality and adequacy of the fitness function were tested experimentally. Experimental results show that the proposed fitness function is better than existing fitness functions for each algorithm used in the test.

The paper “Expected Utility Based Decision Making under *Z*-Information and Its Application” by R. R. Aliev et al. presents decision making under *Z*-information based on direct computation over *Z*-numbers. *Z*-numbers based formalization of information represents a natural language- (NL-) based value of a variable of interest in line with the related NL-based reliability. The approach utilizes expected utility paradigm and is applied to a benchmark decision problem in economics.

The paper “An Intelligent Model for Pairs Trading Using Genetic Algorithms” by C.-F. Huang et al. presents the solution of pairs trading problem using genetic algorithms (GA). In this problem, the pairs of stocks are bought and sold in pair combinations for arbitrage opportunities. The results showed that the GA-based models are generating robust models to tackle the dynamic characteristics in the financial application and provide an effective solution to pairs trading for investment in practice.

The paper “An Enhanced Differential Evolution with Elite Chaotic Local Search” by Z. Guo et al. presents an enhanced differential evolution with elite chaotic local search (DEECL) to solve complex optimization problems. The algorithm utilizes a chaotic search strategy based on the heuristic information from the elite individuals to promote the exploitation power. The experimental results using classical test functions show that DEECL is very competitive on the majority of the test functions.

The paper “A Multiuser Manufacturing Resource Service Composition Method Based on the Bees Algorithm” by Y. Xie et al. presents multiuser resource service composition (RSC) for modelling of an optimal resource service allocation in current open and service-oriented manufacturing model. The model takes into account both subjective and objective quality of service properties as representatives to evaluate a solution. The basic Bees Algorithm is tailored for finding a near-optimal solution to the model. Particular rules are designed for handling the constraints and finding Pareto optimality. In addition, the established model introduces a trusted service set to each user so that the algorithm could start by searching in the neighbour of more reliable service chains (known as seeds) than those randomly generated.

The paper “Phase Response Design of Recursive All-Pass Digital Filters Using a Modified PSO Algorithm” by W.-D. Chang presents a design scheme for the phase response of an all-pass recursive digital filter using a modified PSO (MPSO) algorithm. In the MPSO algorithm, a new adjusting factor is introduced in the velocity updating formula in order to improve the searching ability. The algorithm is applied to design the coefficients of the filter. Two different kinds of linear phase response design examples are illustrated. The obtained results show that the MPSO is superior to the general PSO for the phase response design of the digital recursive all-pass filter.

The paper “Application of Boosting Regression Trees to Preliminary Cost Estimation in Building Construction Projects” by Y. Shin uses a boosting regression tree (BRT) algorithm for estimating the cost at the early stage of a construction project. The boosting approach has attracted attention because of its effective learning algorithm and strong boundaries in terms of its generalization performance. The used BRT model provides additional information such as the importance plot and structure model, which can support estimators in comprehending the decision making process. Consequently, the boosting approach has shown a high performance in cost estimations in a building construction project.

The paper “Predictive Modeling in Race Walking” by K. Wiktorowicz et al. presents the use of linear and nonlinear multivariable models for prediction sports results of athletes practicing race walking. These models are calculated using data collected from race walkers' training events and they are used to predict the result over a 3 km race based on training loads. The paper proposes the nonlinear modifications for linear models and artificial neural networks in order to achieve smaller prediction error. It was shown that the best model is a modified LASSO regression with quadratic terms in the nonlinear part. This model has the smallest prediction error and simplified structure by eliminating some of the predictors.

The paper “Prognostics of Lithium-Ion Batteries Based on Wavelet Denoising and DE-RVM” by C. Zhang et al. presents a novel battery capacity prognostics approach in order to estimate the remaining useful life (RUL) of lithium-ion batteries. Wavelet denoising is performed twice with different thresholds in order to weaken the strong noise and remove the weak noise. Relevance vector machine (RVM) improved by differential evolution (DE) algorithm is utilized to estimate the battery RUL based on the denoised data. An experiment involving battery 5 capacity prognostics case and battery 18 capacity prognostics case is conducted and validated that the proposed approach can predict the trend of battery capacity trajectory closely and estimate the RUL precisely.

The paper “Incremental Discriminant Analysis in Tensor Space” by L. Chang et al. presents a machine learning algorithm in tensor space. The algorithm employs tensor representation to carry on discriminant analysis and combine incremental learning to alleviate the computational cost. The experiments on facial image detection have shown that the algorithm improves the performance of the system compared with other algorithms and reduces the computational issues apparently.

Although the above papers do not completely cover all the aspects of fusion of computational intelligence techniques, they provide important issues and the benefits of practical applications of computational intelligence techniques in engineering and science.

